# Long-term projections of economic growth in the 47 prefectures of Japan: An application of Japan shared socioeconomic pathways

**DOI:** 10.1016/j.heliyon.2021.e06412

**Published:** 2021-03-08

**Authors:** Keita Honjo, Kei Gomi, Yuko Kanamori, Kiyoshi Takahashi, Keisuke Matsuhashi

**Affiliations:** a Global Warming Countermeasures Group, Center for Environmental Science in Saitama (CESS), 914 Kamitanadare, Kazo, Saitama, 347-0115, Japan; b Center for Social and Environmental Systems Research, National Institute for Environmental Studies (NIES), 16-2 Onogawa, Tsukuba, Ibaraki, 305-8506, Japan

**Keywords:** Economic projection, Socioeconomic scenario, Population aging and decline, Productivity, Regional disparity

## Abstract

Assessing climate change impacts on local communities is an urgent task for national and subnational governments. The impact assessment requires socioeconomic scenarios, including a long-term outlook for demographic and economic indices. In Japan, the National Institute for Environmental Studies developed the Japan Shared Socioeconomic Pathways (JPNSSPs) and presented regional population scenarios corresponding to five different storylines. However, there exists no quantitative information about changes in local economies under the population scenarios. This study examines the economic activities in Japan's 47 prefectures using statistical models and calculates changes in the major economic indices (e.g., production, capital stock, and labor population) until 2100. The economic projection is based on ten socioeconomic scenarios generated from the JPNSSP population scenarios and original productivity scenarios. The economic projection results clearly show that Japan's population aging and decline have catastrophic impacts on national and subnational economies. Even in the most optimistic scenario, assuming a massive influx of immigrants and fast productivity growth, the GDP growth rate becomes negative in the 2090s. In the most pessimistic scenario, the GDP growth rate becomes negative in 2028 and continues to decline. As a result, Japan's GDP decreases to the level of the 1970s by 2100. The improvement of productivity cannot offset the GDP shrink caused by demographic changes. Furthermore, the population aging and decline accelerate the wealth concentration in urban areas. The Theil index, calculated using the economic projection results, shows increasing trends in all the scenarios. Tokyo's presence in Japan's economy will continue to increase throughout this century. Meanwhile, Kanagawa and Saitama, which belong to the top five prefectures in terms of economic production, may lose their positions. The Tohoku region, already suffering from population decline, will face severe economic stagnation. Our findings suggest that the depressing future is inevitable unless Japan overcomes the population aging and decline.

## 1 Introduction

Assessing climate change impacts on local communities is an urgent task for national and subnational governments (IPCC, 2014). In December 2018, the Government of Japan enforced the Climate Change Adaptation Act, which strongly encourages all subnational governments (47 prefectures and 1,724 municipalities) to create long-term plans for adapting to climate change (Ministry of the Environment, 2018). Climate researchers are working on the impact assessment of regional climate change in cooperation with policymakers, firms, and other stakeholders (Hara and Shimada, 2017; Masutomi et al., 2019). However, they are now facing a common problem in that the socioeconomic scenarios are not available for local communities.

Long-term scenarios of climate and socioeconomic conditions are necessary for the impact assessment. For climate conditions, Japan's research institutes conducted high-resolution simulations based on scientifically-tested models and published the calculation results as regional climate scenarios (Fujita et al., 2019; Nakagawa et al., 2020). However, there exist no shared socioeconomic scenarios for local communities. Individual researchers are forced to project population dynamics, economic growth, and land-use changes using their own methods. As the impact assessment results based on different assumptions cannot be compared, the lack of shared socioeconomic scenarios confuses policymakers in subnational governments. In national-scale climate studies, the Shared Socioeconomic Pathways (SSPs) (Riahi et al., 2017; IIASA, 2018) are widely used as socioeconomic scenarios, which help improve the communication between researchers and policymakers (Rohat et al., 2019; Tamura et al., 2019; Franzke and Czupryna, 2020). To accelerate climate policies in local communities, we need to develop regional socioeconomic scenarios like the SSPs.

Only a few studies explore subnational-scale socioeconomic scenarios based on the SSPs (Absar and Preston, 2015; Kebede et al., 2018; Chen et al., 2020; Chae et al., 2020). For instance, Chae and co-workers (Chae et al., 2020) created three national-scale scenarios by interpreting the SSPs in the South Korean context. They computed changes in population, economic production, and land use by the end of the century and quantitatively described the future of local communities under the national-scale scenarios. The economic projection is based on a production function that considers the link between population dynamics and economic growth. The National Institute for Environmental Studies (NIES) created the Japan Shared Socioeconomic Pathways (JPNSSPs) with five different storylines (Chen et al., 2020). The research team made long-term population projections for all the municipalities and published the calculation results online (Gomi et al., 2020; NIES, 2020). Chen and co-workers also showed Japan's GDP projections but did not consider the link between population dynamics and economic growth. Moreover, they did not present quantitative information about changes in local economies under the population scenarios.

This study examines the economic activities in Japan's 47 prefectures using statistical models and calculates changes in the major economic indices (e.g., production, capital stock, and labor population) until 2100. The research purpose is to build a regional economic dataset consistent with the JPNSSP population scenarios (Chen et al., 2020; Gomi et al., 2020; NIES, 2020). We focus on three points in the economic projection. First is the relationship between population aging and economic growth. Japan is facing a population decline caused by rapid population aging (Heller, 2016; Liu and Westelius, 2016). The rate of the population aged 65 and over (65+) was 12.1% in 1990, but it increased to 26.6% in 2015 (IPSS, 2020). Japan's total population is projected to decrease from 127,095 thousand persons to 88,077 thousand persons between 2015 and 2065 (IPSS, 2017). Many studies suggest that population aging has negative impacts on economic growth (Bloom et al., 2010; Anderson et al., 2014; Liu and Westelius, 2016; Han, 2019). Our regional economic model can evaluate the impacts of labor supply changes on industrial and commercial production.

Second is the relationship between productivity and economic growth. A popular index for productivity is total factor productivity (TFP), which is defined as the proportion of output (production) to the total input (labor, capital, and other resources). If the total input is constant, higher TFP leads to higher production. TFP growth is an important factor behind economic growth. TFP growth slowed after Japan's bubble economy collapsed at the beginning of the 1990s, resulting in low GDP growth rates (Hayashi and Prescott, 2002; Caballero et al., 2008; Esteban-Pretel et al., 2010; Liu and Westelius, 2016). Long-term TFP projection is difficult because it is influenced by demographic dynamics, macroeconomic policies, and technological changes. This study extends the Cobb-Douglas production function to a dynamic linear model (Petris et al., 2009; Durbin and Koopman, 2012) and expresses the time variation of log-scale TFP as a Gaussian random walk. This approach enables us to estimate the magnitude of uncertainty in the historical changes in TFP. The model estimation result helps create TFP scenarios for local communities.

Third is the economic disparities between local communities. Japan has experienced an overconcentration of population in urban areas, especially in the Greater Tokyo Area (GTA). The GTA consists of only four prefectures (Saitama, Chiba, Tokyo, and Kanagawa), but its total population reached 36.13 million in 2015, which accounted for 28.4% of Japan's population (Statistics Bureau of Japan, 2020). The nominal gross regional product (GRP) was 181.54 trillion JPY in 2015, which was equal to 33.2% of Japan's GDP (Cabinet Office of Japan, 2020a). According to the population projection by IPSS (2017), population aging proceeds in all the prefectures, but the population decline in the GTA is slower than in other areas. As a result, the GTA's population share continues to increase until 2045, which may reinforce the wealth concentration. This study calculates the Theil index (Bourguignon, 1979; Cowell, 2011; Mishra and Ayyub, 2019) from the economic projection results and quantifies regional economic disparities under JPNSSPs.

This paper is structured as follows. Section 2 introduces tools for economic projection: the model structure, model equations, input data, population scenarios, and TFP scenarios. Section 3 summarizes the results of the model estimation and economic projections. We also discuss the long-term outlook for national and subnational economies under JPNSSPs. Finally, we conclude by pointing out the limitations of our approach. The R codes and data files used for the economic projection are available at the Mendeley Data (Honjo, 2021).

## 2 Materials and methods

### 2.1 Model structure

**Figure 1:**
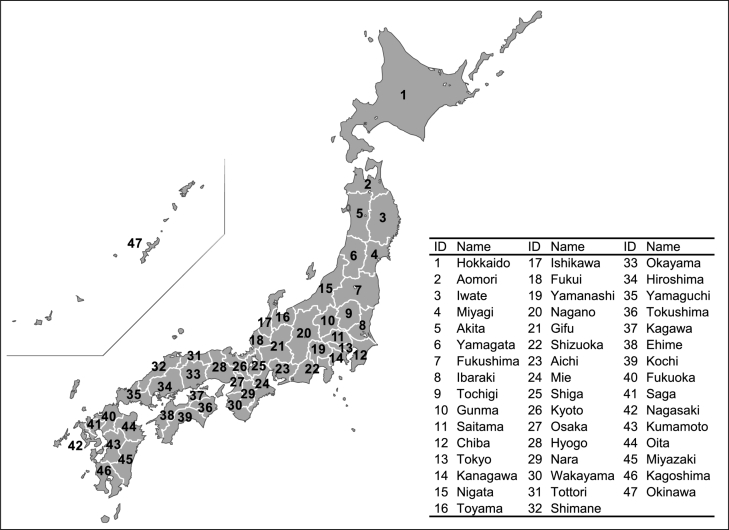
47 prefectures of Japan. No political assertion on Japan's territory is intended.

**Figure 2:**
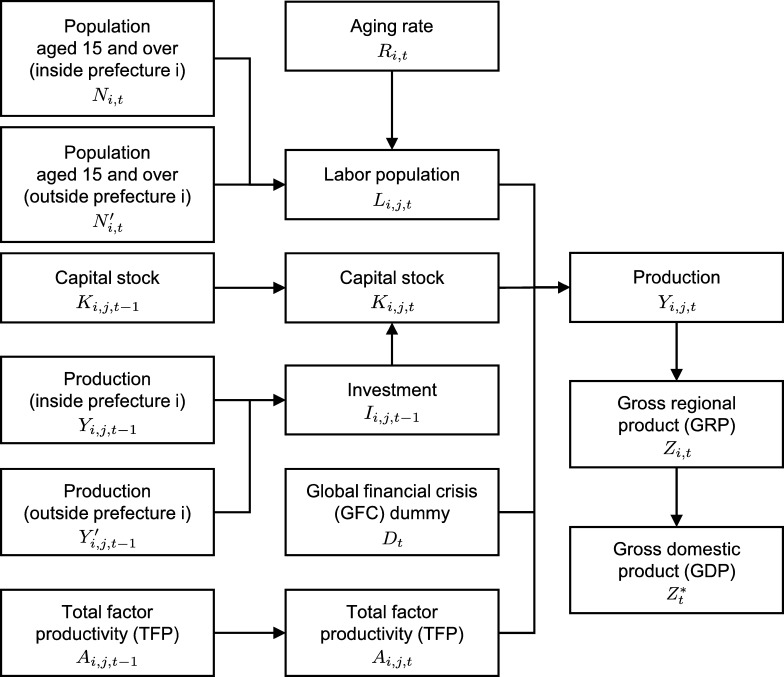
Structure of the regional economic model. The index *i* ∈ {1,2,…,47} denotes a prefecture ID, and the index *j* ∈ {industrial,commercial} denotes a sector.

As the first step of the economic projection, we develop statistical models for economic activities in Japan's 47 prefectures (Fig. 1). Each prefecture's model estimates industrial and commercial production from input data. Fig. 2 shows the model structure. Our model uses the Cobb-Douglas production function, which has total factor productivity (TFP), capital stock, labor population, and the 2008–2009 global financial crisis (GFC) dummy as explanatory variables. Unlike the standard approach (Cobb and Douglas, 1928; Keen et al., 2019), we extend the production function to a dynamic linear model (DLM) (Petris et al., 2009; Durbin and Koopman, 2012) and express the time variation of log-scale TFP as a Gaussian random walk process (see Section 2.2). The DLM is a linear regression model with time-varying parameters suitable for estimating unobservable quantities such as TFP (Arisoy and Ozturk, 2014; Takeda et al., 2016; Honjo et al., 2018). The capital stock in each year is estimated from the previous year's capital stock and investment. The investment depends on the production inside and outside each prefecture. The labor population is estimated from the population aged 15+ and aging rate. The population outside each prefecture is also an explanatory variable because the labor population includes workers from other prefectures. The aging rate is the proportion of the population aged 65+ to the population aged 15+.

### 2.2 Model equations

Here, we show three equations constituting the regional economic model: a dynamic production function, capital stock function, and labor population function. As shown by Fig. 2, variables and parameters have prefecture and sector indices (*i* and *j*). For readability, these indices will not be shown hereafter.

First, the dynamic production function is written as

(1) $$\log Y_{t}=\log A_{t}+\alpha_{1}\log K_{t}+(1-\alpha_{1})\log L_{t}+\alpha_{2}D_{t}+v_{t},$$

(2) $$\log A_{t}=\log A_{t-1}+w_{t},$$

where $Y_{t}$ is production, $A_{t}$ is TFP, $K_{t}$ is capital stock, $L_{t}$ is labor population, $D_{t}$ is the GFC dummy, $v_{t}∼N(0,V)$ is an observation error, and $w_{t}∼N(0,W)$ is a state error. There are five unknown parameters: $A_{t}>0$, $\alpha_{1}\in [0,1]$, $\alpha_{2}\leq 0$, V>0, and W>0. TFP is constant in the classic production function, but is a time-varying parameter in the dynamic production function. The production increases as the TFP, capital stock, and labor population increase. The GFC dummy captures a fall in production due to the GFC.

Second, the growth of capital stock is written as

(3) $$K_{t}=(1-b_{0})K_{t-1}+I_{t-1},$$

where $I_{t}$ is investment and $b_{0}\in [0,1]$ is the depreciation rate. As each prefecture's investment depends on production inside and outside the prefecture ($Y_{t}$ and $Y_{t}^{\prime }$), we have

(4) $$I_{t}=b_{1}+b_{2}Y_{t}+b_{3}Y_{t}^{\prime },$$

where $b_{1}\in (-\infty ,\infty )$, $b_{2}\geq 0$, and $b_{3}\in (-\infty ,\infty )$ are unknown parameters. From equation (3), we have

(5) $$K_{t}=(1-b_{0})K_{t-1}+b_{1}+b_{2}Y_{t-1}+b_{3}Y_{t-1}^{\prime }.$$

For the capital stock function, we use the modified version of equation (5):

(6) $$K_{t}=\beta_{0}+\beta_{1}K_{t-1}+\beta_{2}Y_{t-1}+\beta_{3}Y_{t-1}^{\prime }+v_{t}^{K},$$

where $v_{t}^{K}∼N(0,V^{K})$ is an error term. There are five unknown parameters: $\beta_{0}\in (-\infty ,\infty )$, $\beta_{1}\in [0,1]$, $\beta_{2}\geq 0$, $\beta_{3}\in (-\infty ,\infty )$, and $V^{K}>0$. The capital stock decreases at a constant rate every year. At the same time, the investment determined by the production inside and outside each prefecture increases the capital stock.

Third, the labor population function is written as

(7) $$\log L_{t}=\gamma_{0}+\gamma_{1}\log N_{t}+\gamma_{2}\log N_{t}^{\prime }+\gamma_{3}\log R_{t}+v_{t}^{L},$$

where $N_{t}$ is the population aged 15+ inside prefecture *i*, $N_{t}^{\prime }$ is the population aged 15+ outside prefecture *i*, $R_{t}$ is the aging rate, and $v_{t}^{L}∼N(0,V^{L})$ is an error term. There are five unknown parameters: $\gamma_{0}\in (-\infty ,\infty )$, $\gamma_{1}\geq 0$, $\gamma_{2}\geq 0$, $\gamma_{3}\in (-\infty ,\infty )$, and $V^{L}>0$. The labor population increases as the population inside and outside each prefecture increases. No restriction is imposed on the effect of the aging rate.

### 2.3 Input data

Historical data on production, capital stock, and labor population are from the Regional-Level Japan Industrial Productivity (R-JIP) Database 2017 (Tokui et al., 2013; RIETI, 2018). Population data are from Statistics Bureau of Japan (2020). The economic data cover the period from 1970 to 2012, while the population data cover the period from 1975 to 2018. Therefore, we use the data between 1975 and 2012 (n=38) for the model estimation.

### 2.4 Model estimation methods

The estimation of the dynamic production function consists of two steps. First, we compute the maximum likelihood estimates of the error variances (*V* and *W*). As the model estimation result depends on the initial parameters, we search for a good starting condition using a simple grid-search method. Second, we input the estimated error variances to the model equations and compute the regression coefficients with the Kalman smoother (Petris et al., 2009; Durbin and Koopman, 2012). Table 1 lists the detailed calculation conditions.

**Table 1:** Conditions for estimating the dynamic production function parameters.

Software	R 4.0.3
Library	dlm 1.1-5 (Petris et al., 2009; Petris, 2010)
Parameter estimation methods	
Error variances	Maximum likelihood estimation
Regression coefficients	Kalman smoother
Initial values of error variances	$(V,W)\in {\left\{e^{-15},e^{-14},\ldots ,e^{-5}\right\}}^{2}$

Unlike the dynamic production function, the capital stock function and labor population function are static regression models. We estimate the regression coefficients using the elastic net (Zou and Hastie, 2005; Hastie et al., 2009) and check whether the explanatory variables contribute to the prediction of the response variables. The elastic net, which is a combination of the ridge regression and LASSO, automatically reduces the regression coefficients of the insignificant explanatory variables to zero. This estimation method is applicable to a case where the explanatory variables are correlated with each other. The regression coefficient estimates are obtained by solving the following problem:

(8) $${\mathrm{argmin}}_{\beta }\left[\frac{{\left\|y-X\beta \right\|}_{2}^{2}}{2n}+\lambda \left(\alpha {\left\|\beta \right\|}_{1}+\frac{(1-\alpha ){\left\|\beta \right\|}_{2}^{2}}{2}\right)\right],$$

where **y** is an output vector, **X** is an input matrix, *β* is a regression coefficient vector, n>0 is the data size, α∈[0,1] is a mixing parameter, and λ>0 is a regularization parameter. We optimize the hyper-parameters *α* and *λ* with the time-slice (or time-rolling) cross validation (Hyndman and Athanasopoulos, 2018; Kuhn et al., 2020), because our regression models aim to predict time series data. Table 2 lists the detailed calculation conditions.

**Table 2:** Conditions for estimating the hyper-parameters of the elastic net.

Software	R 4.0.3
Libraries	caret 6.0-86 (Kuhn et al., 2020)
	glmnet 4.0-2 (Friedman et al., 2010)
Parameter estimation method	Time-slice cross validation (Hyndman and Athanasopoulos, 2018; Kuhn et al., 2020)
trainControl arguments	
method	“timeslice”
initialWindow	round(0.7 × data size)
horizon	1
fixedWindow	FALSE
Parameter ranges	
Capital stock function	*α* ∈ [0,1], *λ* ∈ [1,1e+06]
Labor population function	*α* ∈ [0,1], *λ* ∈ [1e-06,1]
Number of parameter points	100 × 100 = 10000

### 2.5 Population scenarios

Population scenarios for the 47 prefectures are necessary for the economic projection. This study uses the JPNSSP population scenarios (Chen et al., 2020; Gomi et al., 2020; NIES, 2020). JPNSSPs are long-term socioeconomic scenarios that support the impact assessment of climate change. As of February 2021, population data for all the municipalities for the time period between 2015 and 2100 are available. JPNSSPs consist of five scenarios (JPNSSP1–JPNSSP5), which are based on different demographic assumptions (Table 3). JPNSSP2, called the Reference Road, uses demographic parameters similar to the population projection by IPSS (2018). The other scenarios were created by changing JPNSSP2's demographic parameters.

**Table 3:** Demographic assumptions of JPNSSPs (Chen et al., 2020; Gomi et al., 2020; NIES, 2020).

Scenario	Fertility	Mortality	Migration
JPNSSP1	High^1^	Medium	Medium
JPNSSP2	Medium^2^	Medium	Medium
JPNSSP3	Very low^3^	Medium	Medium
JPNSSP4	Low^4^	Medium	Medium
JPNSSP5	Medium	Medium	High^5^

1 The fertility rate is consistent with the population projection by IPSS (2017) (high fertility and medium mortality).

2 The fertility rate is consistent with the population projection by IPSS (2018) (medium fertility and mortality).

3 The fertility rate converges to 1.0 by 2065.

4 The fertility rate converges to 1.2 by 2065.

5 A net increase of 250 thousand immigrants before 2035 is assumed.

**Figure 3:**
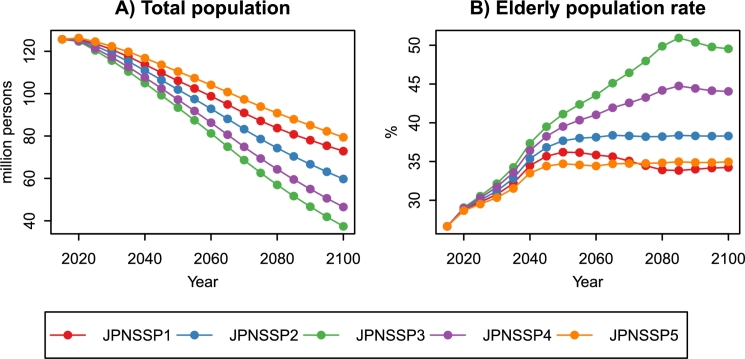
Changes in Japan's total population and the elderly population rate under JPNSSPs, 2015–2100 (Chen et al., 2020; Gomi et al., 2020; NIES, 2020). The elderly population rate is the proportion of the population aged 65+ to the total population.

Fig. 3 shows changes in Japan's total population and elderly population rate (EPR) under JPNSSPs. EPR is the proportion of the population aged 65+ to the total population. In all the scenarios, the total population decreases as EPR increases. The total population ranges from 37.4 million persons in JPNSSP3 to 79.4 million persons in JPNSSP5 in 2100. The JPNSSP3 has a lower fertility rate than the other scenarios. Therefore, EPR is the highest in all the scenarios, and the total population in 2100 is 70.2% lower than the 2015 level. JPNSSP5 has the same fertility rate as JPNSSP2 but assumes a net increase of 250 thousand immigrants before 2035. As a result, the EPR increase and total population decline are curbed.

**Figure 4:**
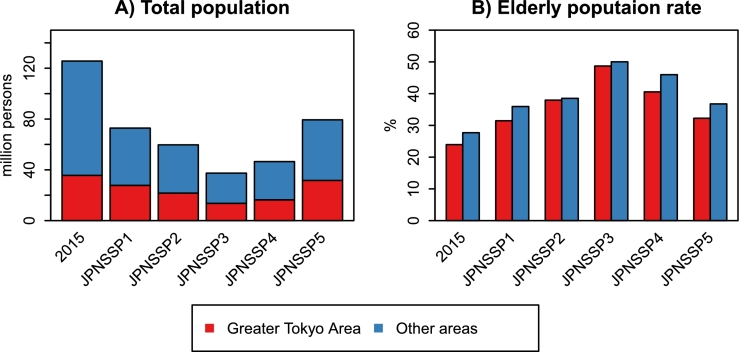
Total population and elderly population rates inside and outside the Greater Tokyo Area. The data for 2015 are from the Census. The other data are the projected values for 2100 based on JPNSSPs (Chen et al., 2020; Gomi et al., 2020; NIES, 2020).

Population aging affects not only the population size but also the population distribution. Fig. 4 shows the total population and EPRs inside and outside the GTA. The data for 2015 and 2100 are compared. Population aging and decline proceed even in the GTA, but the demographic changes are slower than in the other areas. The GTA's population share for 2100 ranges from 35.3% in JPNSSP4 to 39.8% in JPNSSP5, which is higher than the 2015 level (28.4%). All JPNSSPs predict that the population concentration in GTA will continue throughout this century.

### 2.6 TFP scenarios

**Figure 5:**
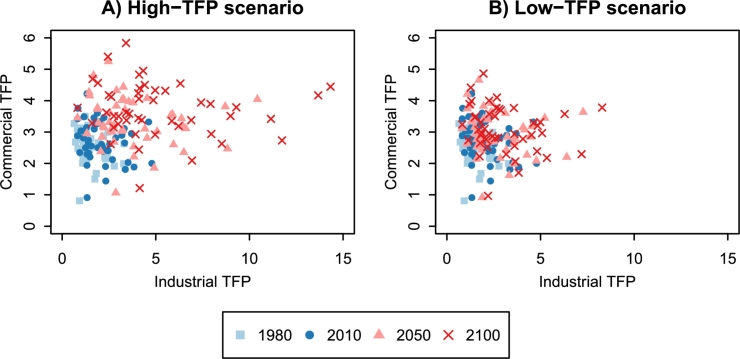
Growth of industrial and commercial TFP in the 47 prefectures of Japan. The data for 1980 and 2010 are model estimates, and the data for 2050 and 2100 are projected values. The high-TFP (low-TFP) scenario assumes that each prefecture's log-scale TFP passes the 95% (75%) point of the probability distribution during the period 2013–2100.

TFP scenarios for the 47 prefectures are also necessary for the economic projection. We created two TFP scenarios based on historical changes: high-TFP and low-TFP. As shown by equation (2), the dynamic production function assumes that log-scale TFP follows a Gaussian random walk. Using the state error variance *W*, which indicates the magnitude of uncertainty, we can calculate the probability distribution of TFP as follows: the dynamic production function gives the estimate of log-scale TFP for 2012. By equation (2), the probability distribution of log-scale TFP for 2013 is written as $N(\log A_{2012},W)$. Generally, the probability distribution of log-scale TFP for year $t_{0}>2012$ is written as $N(\log A_{2012},(t_{0}-2012)W)$. The high-TFP (low-TFP) scenario assumes that each prefecture's TFP passes the 95% (75%) point of the probability distribution during the period 2013–2100. Fig. 5 shows the growth of the industrial and commercial TFP in the 47 prefectures.

### 2.7 Inequality index

This study aims to evaluate the impacts of population aging and decline on regional economic disparities. In welfare economics, a variety of indices for quantifying income inequalities have been proposed: the Gini, Atkinson, and Theil indices (Atkinson, 1970; Bourguignon, 1979; Cowell, 2011; Mishra and Ayyub, 2019). We use the Theil index, which is mathematically equivalent to the Shannon entropy (Shannon, 1948). The Theil index is calculated as

(9) $$T_{t}=\frac{1}{47}\sum_{i=1}^{47}\frac{Z_{i,t}}{{\overset{¯}{Z}}_{t}}\log\left(\frac{Z_{i,t}}{{\overset{¯}{Z}}_{t}}\right)=\sum_{i=1}^{47}s_{i,t}\log(47s_{i,t}),$$

where ${\overset{¯}{z}}_{t}=\sum_{i=1}^{47}Z_{i,t}/47$ is the average GRP and $s_{i,t}=Z_{i,t}/\sum_{i=1}^{47}Z_{i}$ is the GRP share for prefecture *i*. If all the prefectures have equal GRP, the Theil index takes the minimum value of zero, which implies complete equality. If a prefecture's GRP is equal to Japan's GDP, the Theil index takes the maximum value of log⁡47≈3.850, which implies complete inequality. An advantage of the Theil index is its decomposability (Bourguignon, 1979; Cowell, 2011). Each prefecture's contribution to regional economic disparities is given by $s_{i,t}\log(47s_{i,t})$.

## 3 Results and discussion

### 3.1 Model estimation results

**Figure 6:**
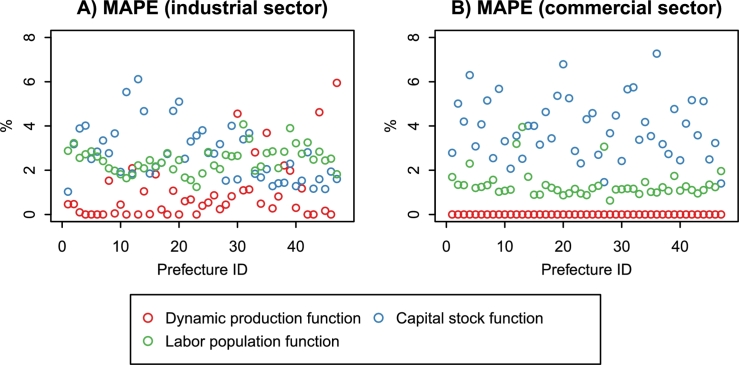
In-sample MAPEs of the model equations.

We estimated the unknown parameters of the dynamic production function, capital stock function, and labor population function using the data from 1975 to 2012. As shown in Fig. 6, the in-sample mean absolute percentage errors (MAPEs) of the model equations are less than 10%. The model equations can explain most of the historical changes in the economic indices. Here, we summarize the estimation results of the regression coefficients.

**Figure 7:**
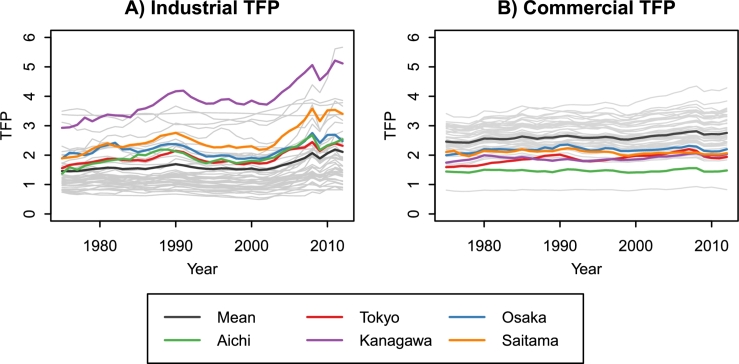
Estimates of industrial and commercial TFP for the 47 prefectures of Japan, 1975–2012. The top five prefectures in GRP (Tokyo, Osaka, Aichi, Kanagawa, and Saitama) are highlighted.

Table 4 presents a statistical summary of the estimated regression coefficients. For each regression coefficient, we calculated the mean and standard deviation (SD) from the estimates of the 47 prefectures. As TFP ($A_{t}$) is a time-varying parameter, we show the complete data in Fig. 7. First, we focus on the regression coefficients other than TFP. $\alpha_{1}$ is the capital elasticity of production. The estimation result reflects the fact that the industrial sector is more capital-intensive than the commercial sector. By equation (1), the production decrease due to GFC is equal to $(\exp(\alpha_{2})-1)\times 100$ [%]. The GFC impact on production was more severe in the industrial sector than the commercial sector. $\beta_{1}$ indicates the speed of capital depreciation. $\beta_{0}$, $\beta_{2}$, and $\beta_{3}$ are associated with investment. $\gamma_{0}$ is an intercept. $\gamma_{1}$ and $\gamma_{2}$ are the elasticities of the labor population with respect to the population inside and outside the prefecture. $\gamma_{3}$ is the elasticity with respect to the aging rate, and its sign is different between the industrial and commercial sectors. The population aging decreased the industrial labor population but increased the commercial labor population.

**Table 4:** Statistical summary of the estimated regression coefficients. For each regression coefficient, the mean and SD were calculated from the estimates of the 47 prefectures.

	Industrial sector		Commercial sector	
	Mean	SD	Mean	SD
Dynamic production function				
*A*_*t*_	See Fig. 7		See Fig. 7	
*α*_1_	0.564	0.179	0.324	0.096
*α*_2_	−0.117	0.052	−0.015	0.007
Capital stock function				
*β*_0_	637413	1108004	917841	1164696
*β*_1_	0.875	0.071	0.840	0.057
*β*_2_	0.038	0.074	0.106	0.211
*β*_3_	0.001	0.002	0.003	0.006
Labor population function				
*γ*_0_	−17.53	13.99	−4.282	5.301
*γ*_1_	1.112	0.696	0.571	0.201
*γ*_2_	0.721	0.818	0.523	0.290
*γ*_3_	−0.934	0.257	0.145	0.075

Second, we focus on the TFP estimates. Fig. 7 shows the historical changes in the industrial and commercial TFP of the 47 prefectures. The top five prefectures in GRP (Tokyo, Osaka, Aichi, Kanagawa, and Saitama) are highlighted. The mean TFP in the industrial sector was flat from 1975 to 2000, but then increased slightly. The temporal mean of industrial TFP in the 2000s was 1.716, which was 9.6% higher than in the 1980s. Kanagawa Prefecture, which has industrial cities such as Yokohama, Kawasaki, and Yokosuka, has maintained the highest TFP since the 1980s. In 2011 and 2012, however, Miyagi Prefecture achieved the highest TFP. Miyagi is one of the prefectures damaged by the 2011 Tohoku earthquake and tsunami. Public investment in the affected areas may have pushed up Miyagi's industrial TFP.

The mean TFP in the commercial sector was higher than in the industrial sector but remained flat over the period. The temporal mean of commercial TFP in the 2000s was 2.693, which was 4.6% higher than in the 1980s. The commercial sector in the Tokyo Metropolis is an essential part of Japan's economy, and its production (67.47 trillion JPY in 2010) accounts for 20.9% of the domestic commercial production. However, the temporal mean of Tokyo's commercial TFP in the 2000s was lower than that of 20 prefectures. This result suggests that the development of Tokyo's commercial sector has been driven by the concentration of capital stock and labor population rather than productivity growth.

### 3.2 Economic projection results

**Figure 8:**
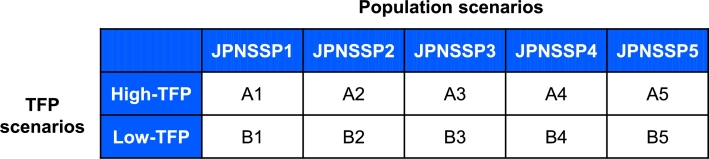
Ten combined scenarios for economic projection.

Here, we show the economic projection results based on the regional economic model and socioeconomic scenarios. As illustrated in Fig. 8, we created ten socioeconomic scenarios (A1–A5 and B1–B5) by combining five population scenarios (JPNSSP1–JPNSSP5) and two TFP scenarios (high-TFP and low-TFP). See Sections 2.5 and 2.6 for the details of the population and TFP scenarios. We input the combined scenarios into the regional economic model and calculated changes in the economic indices by 2100.

#### 3.2.1 National-scale summary

**Figure 9:**
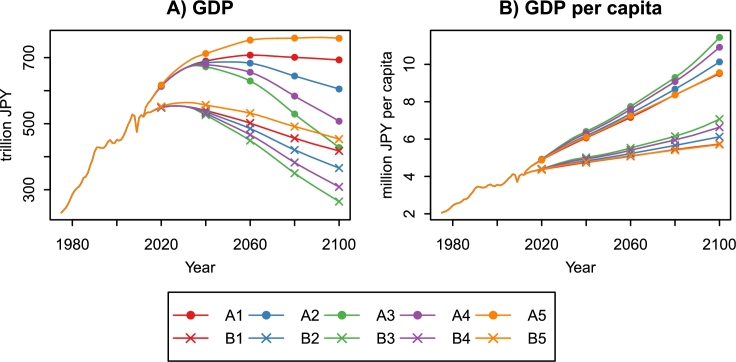
Japan's GDP and GDP per capita under ten combined scenarios, 1975–2100. Economic value is measured in constant 2000 JPY. The data between 1975 to 2012 are from the R-JIP Database 2017 (Tokui et al., 2013; RIETI, 2018).

The economic indices of the whole country are obtained by aggregating the projection results for the 47 prefectures. Fig. 9 shows Japan's GDP and GDP per capita between 1975 and 2100. We can immediately find that the population aging and decline have negative impacts on economic growth. In the A5 scenario, which is the most optimistic, the GDP per capita increases 2.37-fold between 2010 and 2100. However, the GDP growth rate becomes lower than 1% in 2025 and remains approximately 0% until 2100. The slowdown of economic growth is apparent in the other scenarios. In the B3 scenario, which is the most pessimistic, the GDP growth rate becomes negative in 2028 and continues to decrease. As a result, Japan's GDP decreases to 264.20 trillion JPY in 2100, equivalent to the level in the 1970s. The GDP shrink also occurs in the A3 scenario, which assumes a higher TFP than B3. The improvement of productivity cannot offset the GDP shrink caused by demographic changes.

**Figure 10:**
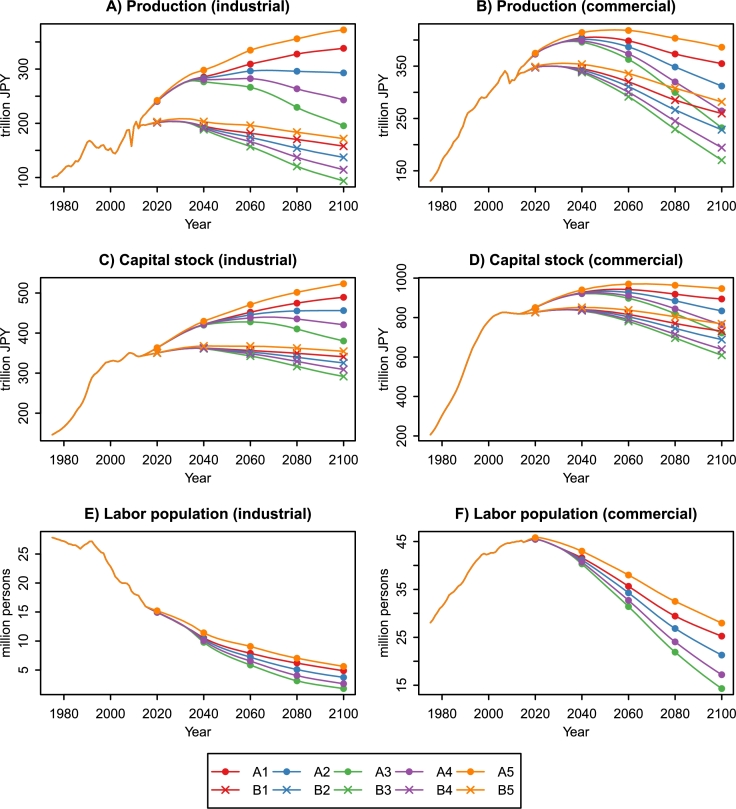
Production, capital stock, and labor population in the industrial and commercial sectors of Japan, 1975–2100. Economic value is measured in constant 2000 JPY. The data between 1975 and 2012 are from the R-JIP Database 2017 (Tokui et al., 2013; RIETI, 2018). The A1–A5 scenarios assume the same population curves as the B1–B5 scenarios.

Fig. 10 shows production, capital stock, and labor population in the industrial and commercial sectors between 1975 and 2100. In recent years, Japan's economic growth has been driven by the development of the commercial sector. Commercial production reached 323.52 trillion JPY in 2010, which was equal to 62.6% of Japan's GDP. The commercial production rate ranges from 50.9% to 54.3% and 62.2% to 64.5% under the A1–A5 and B1–B5 scenarios, respectively, in 2100. The improvement of productivity reduces the difference between industrial and commercial production. This result is interpreted as follows. Both the industrial and commercial sectors face labor force shortages under the population aging and decline. The industrial labor population shows a downward trend since 1975 and is expected to decline by 69.0%–90.0% between 2010 and 2100. The labor population decline in the industrial sector is much faster than in the commercial sector. However, industrial production is more capital-intensive and is less affected by labor supply changes than the commercial sector (Table 4). Moreover, the A1–A5 scenarios predict a significant increase in industrial TFP (Fig. 5), which mitigates the production decline.

#### 3.2.2 Subnational-scale summary

**Figure 11:**
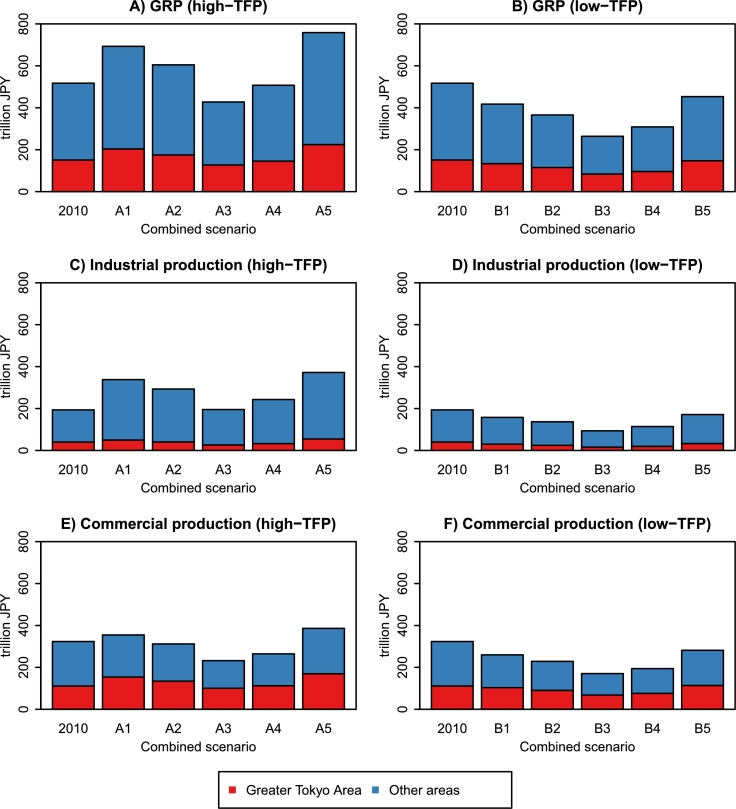
Production projections for the Greater Tokyo Area and other areas. Economic value is measured in constant 2000 JPY. The data for 2010 are from the R-JIP Database 2017 (Tokui et al., 2013; RIETI, 2018). The other data are the projected values for 2100.

We summarize the economic projection results for the 47 prefectures. Fig. 11 shows the production projections for the GTA and other areas. The data for 2010 and 2100 are compared. From this result, we can immediately find that the concentration of wealth in the GTA will continue throughout this century. The GTA's GRP share (GRP / GDP) in 2100 ranges from 28.7% to 29.7% under A1–A5, which is nearly equal to the 2010 level (29.2%). Under B1–B5, the GRP share ranges from 31.1% to 32.5% and exceeds the 2010 level. The shift from low-TFP scenarios (B1–B5) to high-TFP scenarios (A1–A5) slightly mitigates the wealth concentration. As shown in Panels C–F, the GTA's economic activities are mainly led by the commercial sector. The commercial sector is more labor-intensive than the industrial sector and is vulnerable to population aging and decline (Table 4). Moreover, high-TFP scenarios predict that the growth of commercial TFP is much slower than the industrial TFP (Fig. 5). For these reasons, the shift from low-TFP to high-TFP scenarios curbs the increasing economic disparities between the GTA and other areas.

**Figure 12:**
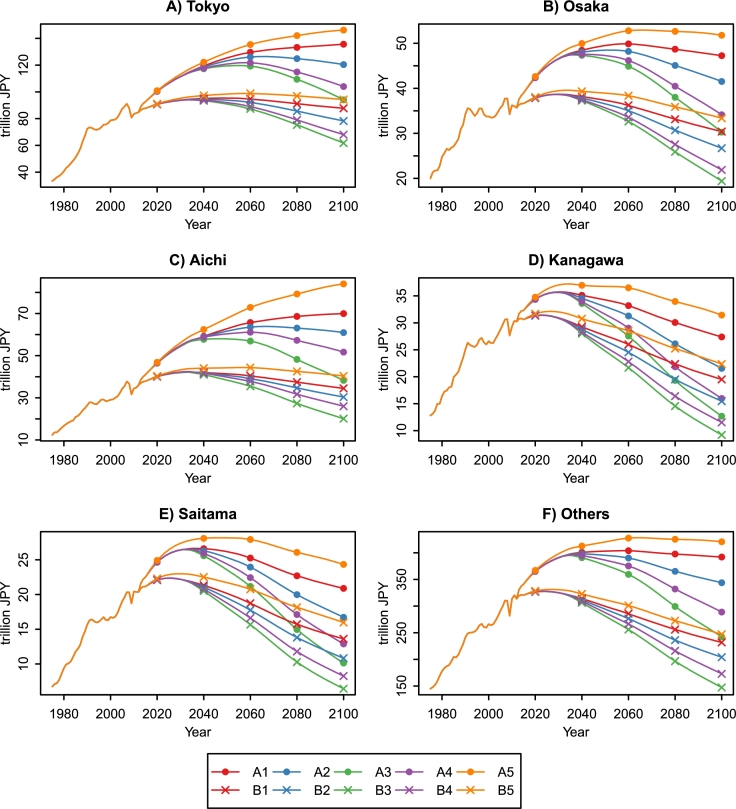
GRP projections for the top five prefectures and others, 1975–2100. Economic value is measured in constant 2000 JPY.

Fig. 12 shows the GRP projections for the top five and other prefectures under the combined scenarios. Sustained economic growth is difficult even in the top five prefectures. The GRP growth rate of Tokyo continues to be positive under A1 and A5. In the most pessimistic scenario (B3), the GRP growth rate becomes negative in 2035. As a result, Tokyo's GRP decreases to the level of the 1980s by the end of the century. Aichi shows a similar trend to Tokyo. Other prefectures face the production decline even in the most optimistic scenario (A5). The GRP growth rates of Osaka, Kanagawa, and Saitama become negative in 2067, 2035, and 2050, respectively. The GRP decreases in these prefectures are more severe than in Tokyo and Aichi.

**Figure 13:**
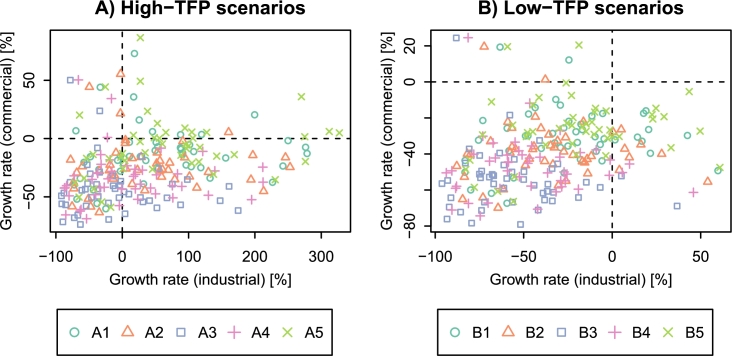
Growth rates of industrial and commercial production in the 47 prefectures of Japan. The growth rates were calculated from the data for 2010 and 2100.

Fig. 13 shows the growth rates of industrial and commercial production in the 47 prefectures. The growth rates were calculated from the data for 2010 and 2100. In high-TFP scenarios (A1–A5), many prefectures increase their industrial production but decrease the commercial production. There are a small number of cases where both the industrial and commercial sectors have positive growth rates (e.g., Tokyo, Aichi, Mie, Shiga, and Osaka under A5). In low-TFP scenarios (B1–B5), there exists no case where the industrial production and commercial production increase simultaneously. Most of the prefectures experience the shrink of industrial and commercial production. The B3 scenario brings severe economic stagnation to the Tohoku region, already suffering from population decline. By the end of the century, GRP in Aomori, Iwate, Akita, Yamagata, and Fukushima decreases by more than 75% from the 2010 levels.

**Figure 14:**
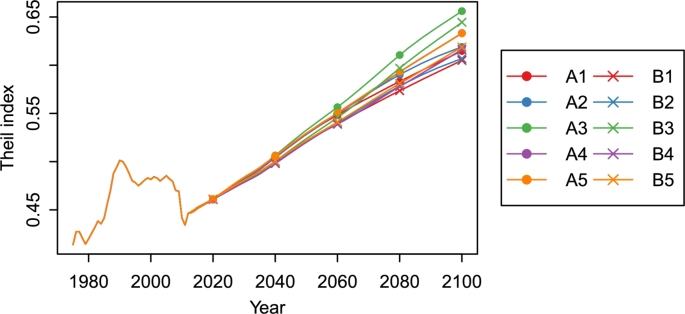
Theil indices calculated from GRP projections for the 47 prefectures of Japan, 1975–2100.

The economic projection results indicate that the slowdown of economic growth due to the population aging and decline occurs in all the prefectures. The concentration of wealth in urban areas (e.g., Tokyo, Osaka, and Aichi) seems to be maintained. We calculated the Theil index from the GRP projections to quantify the regional economic disparities (Fig. 14). The Theil index shows increasing trends in all the combined scenarios, which means that the regional economic disparities increase with the population aging and decline. The Theil index in 2100 is the highest under A3 and the lowest under B1. However, the differences in the combined scenarios do not affect the long-term trend of the Theil index.

**Figure 15:**
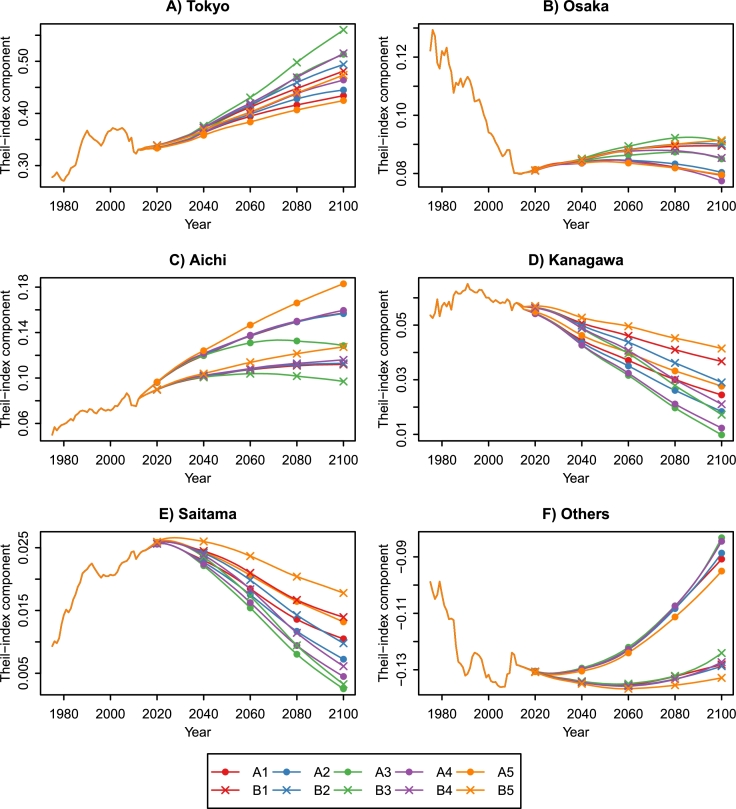
Theil index components for the top five and other prefectures, 1975–2100.

Finally, we quantified the prefectures' contributions to the increased economic disparities using the decomposability of the Theil index (see Section 2.7). Fig. 15 shows the Theil index components of the top five and other prefectures between 1975 and 2100. Tokyo's component is the highest in all the prefectures and shows increasing trends in all the combined scenarios. Tokyo's presence in Japan's economy will continue to increase throughout this century. Aichi's component also shows increasing trends, but turns into decreasing trends under A3 and B3. Osaka's component, which has decreased since 1975, turns into increasing trends in some scenarios. However, it is difficult for Osaka to recover the past economic presence. Kanagawa's and Saitama's components show decreasing trends in all the scenarios. The aggregate component of the other prefectures shows U-shaped curves because several prefectures (e.g., Mie, Shiga, and Hiroshima) increase their GRP shares. Kanagawa and Saitama, which belong to the top five prefectures, may lose their positions and be replaced with Mie, Shizuoka, and Hyogo by the end of the century (Table 5).

**Table 5:** Top five prefectures in GRP at the end of the century.

Scenarios	GRP rank				
	1st	2nd	3rd	4th	5th
2010	Tokyo	Osaka	Aichi	Kanagawa	Saitama
A1–A4	Tokyo	Aichi	Osaka	Mie	Shizuoka
A5	Tokyo	Aichi	Osaka	Mie	Kanagawa
B1–B2	Tokyo	Aichi	Osaka	Kanagawa	Shizuoka
B3	Tokyo	Aichi	Osaka	Shizuoka	Hyogo
B4	Tokyo	Aichi	Osaka	Shizuoka	Kanagawa
B5	Tokyo	Aichi	Osaka	Kanagawa	Hyogo

### 3.3 Limitations

Our approach has three limitations. First, our calculation results do not include the impacts of some severe disasters on Japan's macroeconomic policies. The 2011 Tohoku earthquake and tsunami caused catastrophic damage to the local communities of Miyagi, Iwate, and Fukushima. Moreover, after the nuclear disaster in Fukushima, the shutdown of nuclear power plants resulted in a shortage of electric power supply, which affected economic activities in many parts of Japan (Cho et al., 2016; Kimura and Nishio, 2016; Honjo et al., 2018). This study estimates the model equations using the data from 1975 to 2012. Therefore, it is difficult to analyze the earthquake impacts in detail. For the same reason, we do not consider the economic stagnation caused by the COVID-19 pandemic (Cabinet Office of Japan, 2020b). Additional historical data for Japan's local economies are necessary to overcome this limitation.

Second, the TFP scenarios used for economic projection underestimate the magnitude of uncertainty because they depend only on already-known information. This study statistically estimates the magnitude of uncertainty from the historical changes in TFP and uses the result to create TFP scenarios. Our approach does not consider several uncertain factors. As assumed in JPNSSP1 (Chen et al., 2020), for example, technological changes driven by the evolution of AI and robots will contribute to higher TFP. However, it is impossible to predict the long-term effects of technological changes on TFP using historical data. Another uncertain factor is the COVID-19 pandemic. Under the state of emergency declared by the Government of Japan, citizens and firms voluntarily reduced their economic activities between April 7 and May 25, 2020 (Cabinet Secretariat of Japan, 2020). Considering the sharp fall in Japan's GDP for the April to June quarter of 2020 (Cabinet Office of Japan, 2020b), TFP may have significantly declined. The government proposed a new lifestyle (e.g., working from home, staggered commuting, and online meetings) and encouraged workers to change Japan's traditional way of working (Cabinet Secretariat of Japan, 2020). This campaign may lead to higher TFP in the long run, but we do not have enough evidence to evaluate its effect. Further studies based on additional data are required to understand the relationship between the COVID-19 pandemic and TFP.

The third is a methodological limitation. The regional economic model is based on statistical methods, and the parameters other than TFP are the constant estimates derived from historical data. In other words, we assume the Business-as-Usual (BAU) case where the variable relationships illustrated by Fig. 2 are stable. The estimated model can explain the economic activities in Japan's 47 prefectures from 1975 to 2012 (Fig. 6). However, future changes in population and technology may affect the model parameters and degrade the prediction performance. The use of DLMs mitigates this limitation. We estimated the time variation of TFP by extending the production function to a DLM (Section 2.2). The same approach can be applied to other parameters, but the simultaneous estimation of multiple time-varying parameters requires extensive input data. Moreover, theoretical analysis is needed to express the parameter dynamics as stochastic processes.

### 3.4 Conclusion

This study examined the economic activities in Japan's 47 prefectures using statistical models and calculated changes in the major economic indices (e.g., labor population, capital stock, and production) until 2100. The research purpose was to build a regional economic dataset consistent with the JPNSSP population scenarios (Chen et al., 2020; Gomi et al., 2020; NIES, 2020). The economic projection is based on the socioeconomic scenarios (A1–A5 and B1–B5) generated from the JPNSSP population scenarios and original productivity scenarios.

The economic projection results clearly show that the population aging and decline have catastrophic impacts on national and subnational economies (Fig. 9). Even in the most optimistic scenario (A5), assuming a massive influx of immigrants and fast productivity growth, the GDP growth rate becomes negative in the 2090s. In the most pessimistic scenario (B3), the GDP growth rate becomes negative in 2028 and continues to decline. As a result, Japan's GDP decreases to the level of the 1970s by 2100. The improvement of productivity cannot offset the GDP shrink caused by demographic changes.

The population aging and decline accelerate the concentration of wealth in urban areas (e.g., Tokyo, Aichi, and Osaka). The Theil index, calculated from the GRP projections for the 47 prefectures, shows increasing trends in all the scenarios (Fig. 14). We also quantified the prefectures' contributions to regional economic disparities using the decomposability of the Theil index (Fig. 15). Tokyo's contribution is the highest in all the prefectures and increases in all the scenarios. In other words, Tokyo's presence in Japan's economy will continue to increase throughout this century. Meanwhile, Kanagawa and Saitama, which belong to the top five prefectures in GRP, may lose their positions. Kanagawa's and Saitama's contributions show decreasing trends and become lower than Mie, Shizuoka, and Hyogo. The Tohoku region, already suffering from population decline, will face severe economic stagnation. In the B3 scenario, GRP in Aomori, Iwate, Akita, Yamagata, and Fukushima decreases by more than 75% between 2010 and 2100. Our findings suggest that the depressing future is inevitable unless Japan overcomes the population aging and decline. The calculation results of this study are available at the Mendeley Data (Honjo, 2021).

Finally, we conclude this paper by showing the direction of future studies. As described in Section 3.3, the development of regional socioeconomic scenarios suffers from the lack of historical data and methodological limitations. This study estimated the regional economic model using the data from 1975 to 2012, provided by the R-JIP Database 2017 (Tokui et al., 2013; RIETI, 2018). We need to update the input data to assess the impacts of the 2011 Tohoku earthquake and the COVID-19 pandemic on Japan's macroeconomic policies. Furthermore, this study assumes the BAU case where the model parameters other than TFP are constant. The combination of statistical and theoretical approaches is necessary to predict the dynamics of local economies.

## Declarations

### Author contribution statement

Keita Honjo: Conceived and designed the experiments; Performed the experiments; Analyzed and interpreted the data; Contributed reagents, materials, analysis tools or data; Wrote the paper.

Kei Gomi, Yuko Kanamori, Kiyoshi Takahashi and Keisuke Matsuhashi: Contributed reagents, materials, analysis tools or data.

### Funding statement

This work was supported by the Environment Research and Technology Development Fund (JPMEERF20182005) of the 10.13039/100014423Environmental Restoration and Conservation Agency of Japan.

### Data availability statement

Data associated with this study has been deposited at Mendeley Data (https://doi.org/10.17632/fpwkb33by5.1).

### Declaration of interests statement

The authors declare no conflict of interest.

### Additional information

No additional information is available for this paper.
